# Synchronous Recruitment of Epigenetic Modifiers to Endotoxin Synergistically Activated Tnf-α Gene in Acute Kidney Injury

**DOI:** 10.1371/journal.pone.0070322

**Published:** 2013-07-30

**Authors:** Karol Bomsztyk, Steve Flanagin, Daniel Mar, Michal Mikula, Ali Johnson, Richard Zager, Oleg Denisenko

**Affiliations:** 1 Department of Medicine, University of Washington, Seattle, Washington, United States of America; 2 Fred Hutchinson Cancer Research Center, Seattle, Seattle, Washington, United States of America; University of Crete, Greece

## Abstract

**Background:**

As a consequence of acute kidney injury (AKI), proximal tubular cells hyperrespond to endotoxin (lipopolysaccharide, LPS) by exaggerated renal Tnf-α Production. This LPS hyperresponsiveness is transcriptionally mediated. The epigenetic pathways that control these responses are unknown.

**Methods/Findings:**

We applied multiplex chromatin immunoprecipitation platform (Matrix ChIP) to explore epigenetic pathways that underlie endotoxin hyperresponsiveness in the setting of preceding unilateral renal ischemia/reperfusion (I/R) in mouse AKI model. Endotoxin exposure after I/R resulted in enhanced transcription, manifested by hyperresponsive recruitment of RNA polymerase II (Pol II) at the Tnf-α gene. At this locus, LPS but not I/R increased levels of Pol II C-terminal domain (CTD) phosho-serine2 &5 and induced dephosphorylation of the transcription-repressive histone H4 phospho-serine-1. In contrast, I/R but not LPS increased the transcription-permissive histone phosphorylation (H3 phospho-serine-10, H3.3 phospho-serine-31) at the Tnf-α gene. In agreement with these observations, I/R but not LPS increased activity of cognate kinases (Erk1/2, Msk1/2 and Aurora A) at the Tnf-α locus. Cross-talk of histone phosphorylation and acetylation synergize to active gene expression. I/R and LPS increased histone acetylation. (H3K9/14Ac, H4K5/8/12/16Ac, H2KA5Ac, H2BK4/7Ac). Levels of some histone acetyltransferases at this gene (PCAF and MOF) were increased by I/R but not by LPS, while others were induced by either I/R or LPS and exhibited endotoxin hyperresponsive patterns (GCN5, CBP and p300). The adaptor protein 14-3-3 couples histone phosphorylation with acetylation, and tethers chromatin modifiers/transcription elongation factors to target genes. Both I/R and LPS increased levels of 14-3-3 and several chromatin/transcription modifiers (BRD4, BRG1, HP-1γ and IKKα) at the Tnf-α gene, all exhibiting endotoxin hyperresponsive recruitment patterns similar to Pol II.

**Conclusions:**

Our results suggest that I/R and LPS differentially trigger phosphorylation (Pol II and histone) and acetylation (histone) epigenetic pathways that interact at the Tnf-α gene to generate endotoxin hyperresponse in AKI.

## Introduction

Classical studies from several laboratories, including Nath et al [1], [2], Rabb et al [3], [4], Star et al [5], [6], [7], Kelly et al [8], [9], Reeves et al [10], [11], [12] and Zager et al [13], [14], [15] have demonstrated that renal inflammation and the resulting efflux of inflammatory mediators into the systemic circulation are critical consequences of acute kidney injury (AKI) [16], [17], [18]. Tumor necrosis factor α (Tnf-α) and other inflammatory mediators play a key role in the pathogenesis of AKI. Zager et al and others have demonstrated that AKI renders the kidney hyperresponsive to endotoxin (lipopolysaccharide, LPS), resulting in enhanced production of Tnf-α [14], [19], [20]. This hyperresponsive state is a consequence of diverse forms of AKI, including ischemia/reperfusion (I/R) [14], [21]. Increased Tnf-α gene transcription is an essential element of this AKI-induced LPS-hypersensitivity [15]. The signaling pathways at chromatin that mediate these transcriptional changes are not known.

Epigenetic processes play a critical role in transcriptional regulation. These processes are driven by changes in covalent modifications of DNA and associated proteins, alterations in chromatin structure, and recruitment of a diversity of signal responsive transcription factors and enzymes. The molecular mechanisms controlling chromatin and transcription are remarkably conserved in organisms as diverse as yeast, flies and mammals [22], [23], [24], [25]. Thus, available information across species and cell types provides a valuable basis to study epigenetic control of Pol II transcription in disease. Defining these signaling pathways at the chromatin level is needed to better understand transcriptional control. Moreover, identification of transcription and epigenetic modifiers engaged at tissue injury-related genes would allow selecting small molecules to target these enzymes to ameliorate AKI.

Guided by other model systems [26], [27], we took advantage of the effectiveness of the multiplex Matrix ChIP platform [28], [29], [30], [31] to provide a first-of-a-kind detailed analysis of epigenetic events encompassing the renal injury-related Tnf-α locus, providing new insights into mechanisms of injury-induced renal hyperresponse to endotoxin exposure [14], [15], [32].

## Results

### Model of Cytokine mRNA Hyperresponse to Endotoxin Following Unilateral Renal I/R

To study mechanisms of renal endotoxin hyperresponsiveness, we chose an I/R model [21] (Fig. S1A) where one kidney of each animal is subjected to ischemia/reperfusion with subsequent treatment with either saline (I/R) or LPS (I/R+LPS). The contralateral kidneys from either saline (CO) or LPS-treated (CO+LPS) groups are used as controls. We define a change as endotoxin hyperresponsive when the levels of Tnf-α measured in I/R+LPS samples are greater than any of the three other samples, CO, I/R and CO+LPS. The graphic and statistical representation of the data and the pattern of hyperresponsiveness are illustrated in Fig. S1B–C.

**Figure 1:**
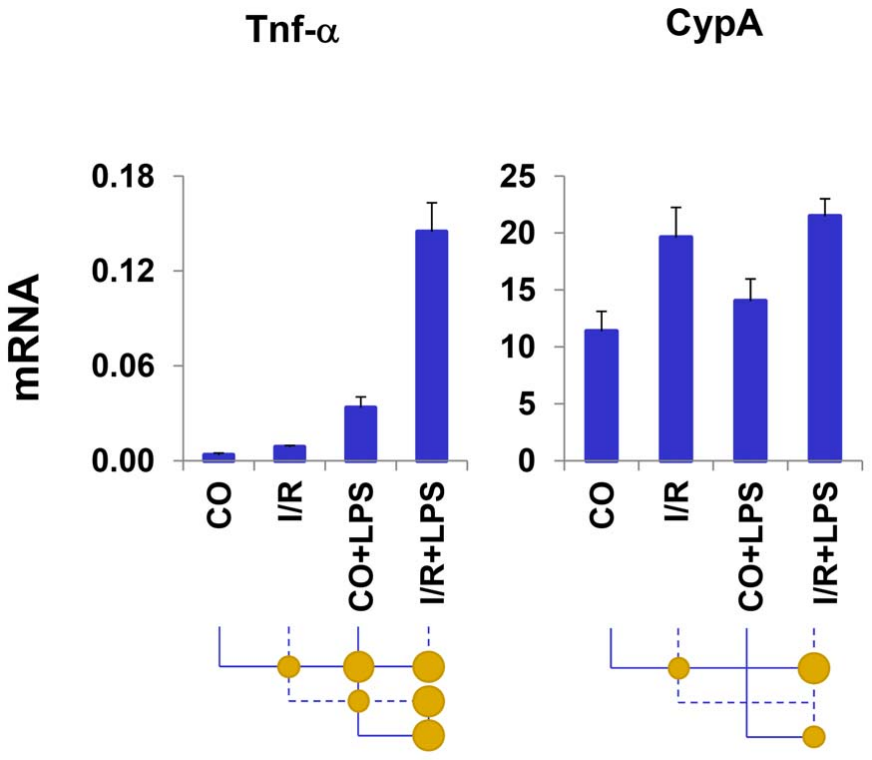
Analysis of renal *Tnf-α* expression following unilateral kidney I/R and LPS injection. Total RNA from mice renal cortex was used in RT reactions with random hexamers. cDNA was used in real time PCR with gene specific primers (Table S1). mRNA level of a given gene in each sample was normalized to βactin transcript. Data are represented as mean±SEM, n = 6 mice in each group.

RT-PCR was used to assess Tnf-α mRNA levels. Cyclophilin A (CypA) gene was used for comparison [33]. In agreement with previous studies, 30 min of unilateral ischemia increased expression of Tnf-α gene following the injury, as assessed 24 hrs later by the mRNA level (I/R vs. CO) [14] (Fig. 1). Two hours following injection of LPS the increase in Tnf-α mRNA levels was greater in the post ischemic (I/R+LPS) compared to I/R and the contralateral kidneys (CO+LPS). LPS-induced Tnf-α mRNA levels were higher than those induced by I/R (CO+LPS>I/R). In contrast to Tnf-α CypA transcript was induced by I/R but not LPS. The response to I/R suggest that in AKI CypA may not be viewed as a classical housekeeping gene. We used these conditions to examine the epigenetic basis for the Tnf-α hyperresponsiveness.

### Pol II and CTD Phosphorylation at Tnf-α Gene in Response to LPS is Exaggerated in the Setting of I/R

**Figure 2:**
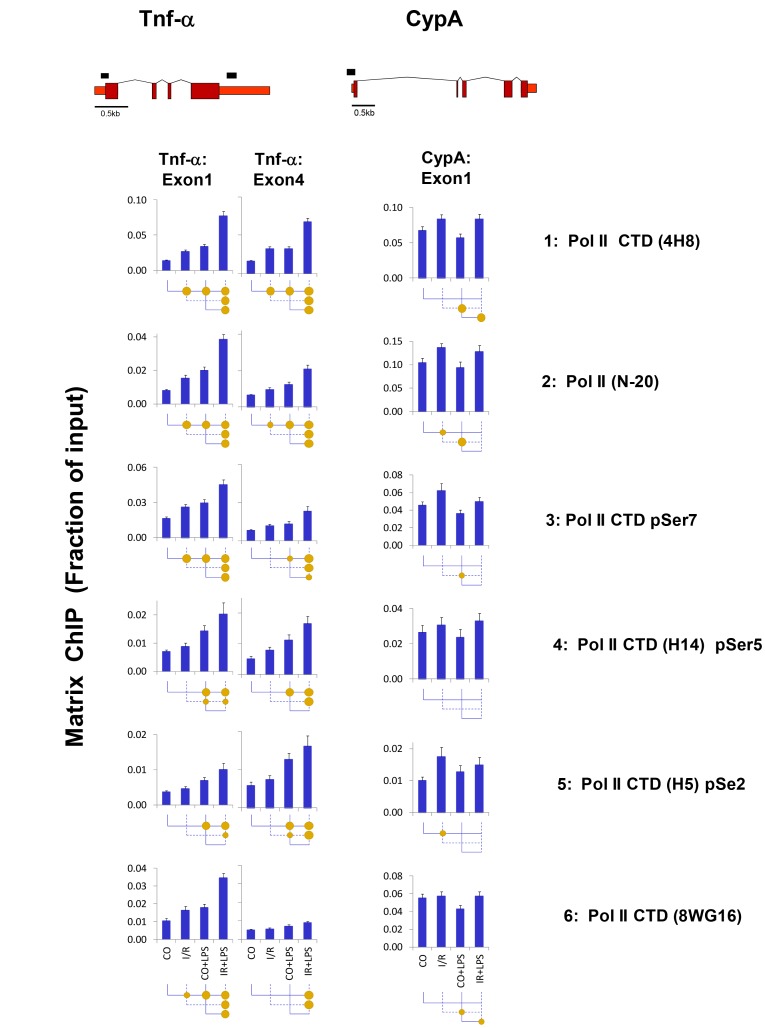
Matrix ChIP analysis of RNA polymerase II (Pol II) *Tnf-α* genes following unilateral kidney I/R and LPS injection. Sheared cross-linked renal cortex chromatin from mice were assayed using antibodies to the Pol II N-terminus and CTD modifications. ChIP DNA were analyzed at the Tnf-α first and last exon and CypA first exon in real-time PCR. Data represent mean ± SEM (6 animals from each group), expressed as fraction of input. Schematic of the genes is shown above the graphs; exons are shown as rectangles (taller and shorter rectangles represent translated and untranslated regions), lines represent introns. Black boxes shows location of the amplicon.

Measurement of Pol II levels along a locus provides means to assess rates of transcription. Analysis of Pol II binding at the Tnf-α gene, estimated by antibodies to the CTD and N-terminal domains, revealed increased levels of the polymerase following either I/R or LPS treatment (Fig. 2). Importantly, there was endotoxin-induced hyper-recruitment in the setting of renal I/R, indicating that transcription is, at least in part, responsible for the exaggerated mRNA response (Fig. 1 & 2). Similar to the CypA mRNA changes (Fig. 1), Pol II levels increased at this locus in response to I/R but not LPS and there was no Pol II hyperresponse at the CypA site in I/R+LPS kidneys. There could be genomic sequences homologous to CypA gene [33]. The CypA PCR primer pair used in ChIP-qPCR yields a single amplicon (one dissociation curve) and in silico PCR analysis of mouse genome identifies only one CypA genomic site (http://genome.ucsc.edu/), thus it is appropriate to use this gene site for comparison with the Tnf-α gene. Injury-induced increases in Pol II levels were seen at both the 5′ and 3′ ends of the Tnf-α locus, indicating that transcriptional initiation plays a role in the transcriptional hyperresponse to endotoxin in this AKI model.

Mammalian Pol II CTD contains 52 heptapeptide repeats (Y_1_S_2_P_3_T_4_S_5_P_6_S_7_), which are sites for modifications that define the state of the polymerase [34]. Phosphorylation of serine 5 is associated with transcriptional initiation while phosphorylation of serine 2 initiates elongation and increases towards the 3′ end of the gene [34]. CTD is also phosphorylated at other sites including serine 7 [35]. Defining kinases that target CTD during AKI may elucidate pathways driving hyperresponsiveness. We used Matrix ChIP and a panel of CTD phosphospecific antibodies to characterize the phosphorylation status of Pol II at the Tnf-α locus. At the 5′ end of the gene, the Pol II pattern of phosphorylated serine 7 of CTD matched injury-induced changes in total polymerase levels (4H8, N-20 and 8WG16), whereas increases in the level of serine 5 and serine 2 phosphorylation in response to I/R alone were smaller than after LPS. These studies indicate that these two treatments engage different signaling pathways to induce transcription of the same gene, where chromatin may account for these differences.

### I/R and LPS Alter Chromatin at the Tnf-α Locus

To define changes in chromatin that may contribute to the endotoxin hyperresponsiveness, we assessed histone marks including those associated with transcription initiation, elongation and repression.

#### Histone post-translational modifications (PTMs) changes associated with transcriptional initiation

**Figure 3:**
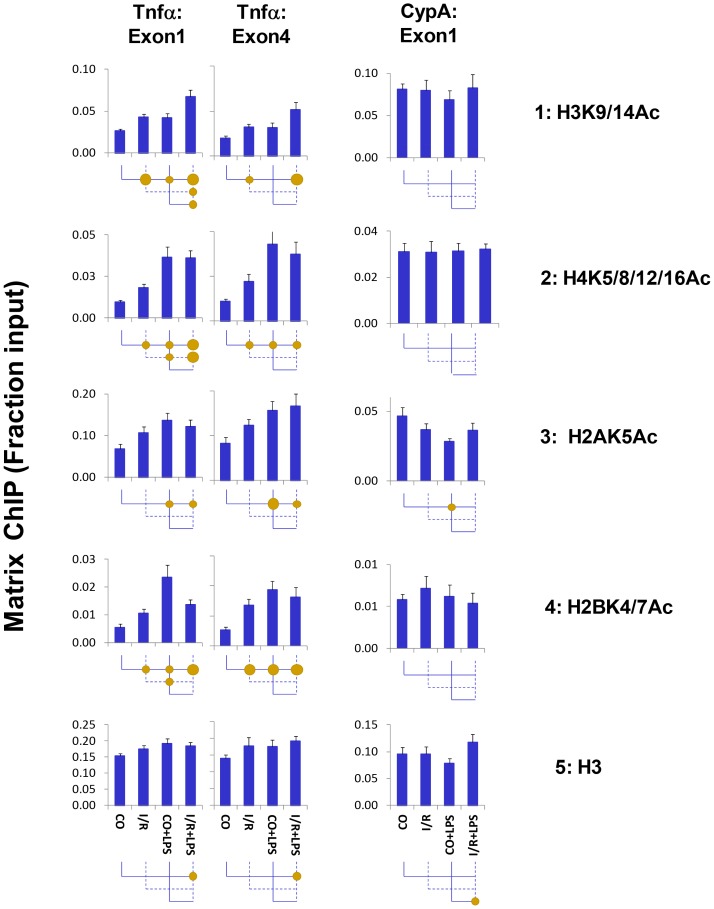
Matrix ChIP analysis of permissive histone acetylation marks at and *Tnf-α* genes following unilateral kidney I/R and LPS injection. Sheared cross-linked renal cortex chromatin from mice were assayed using antibodies to acetylated histones and total histone H3. ChIP DNA were analyzed at the Tnf-α first and last exon and CypA first exon first in real-time PCR. Data represent mean ± SEM (6 animals from each group), expressed as fraction of input.

Histone acetylation can increase in response to extracellular signals to generate permissive chromatin structure [31], [36]. I/R and LPS triggered two types of histone acetylation responses at Tnf-α gene (Fig. 3), i) increased acetylation levels of histone H3 lysines 9 and 14 (H3K9/14Ac) that followed the profile of Pol II changes, and ii) increased acetylation of histone H4 lysines 5,8,12&16 (H4K5/8/12/16), H2A lysine 5 (H2A5Ac), and H2B lysine 4&7 (H2BK4/7Ac) where both I/R and LPS increased the level of acetylation but, unlike H3K9/14Ac, the inducing effect of LPS on the control and I/R kidney was of the same magnitude. Changes in histone H3 were small.

#### Histone PTMs associated with transcriptional elongation

Although several histone PTMs play a role in transcriptional elongation, these processes have not previously been studied in AKI.

#### Histone H3 phosphorylation

**Figure 4:**
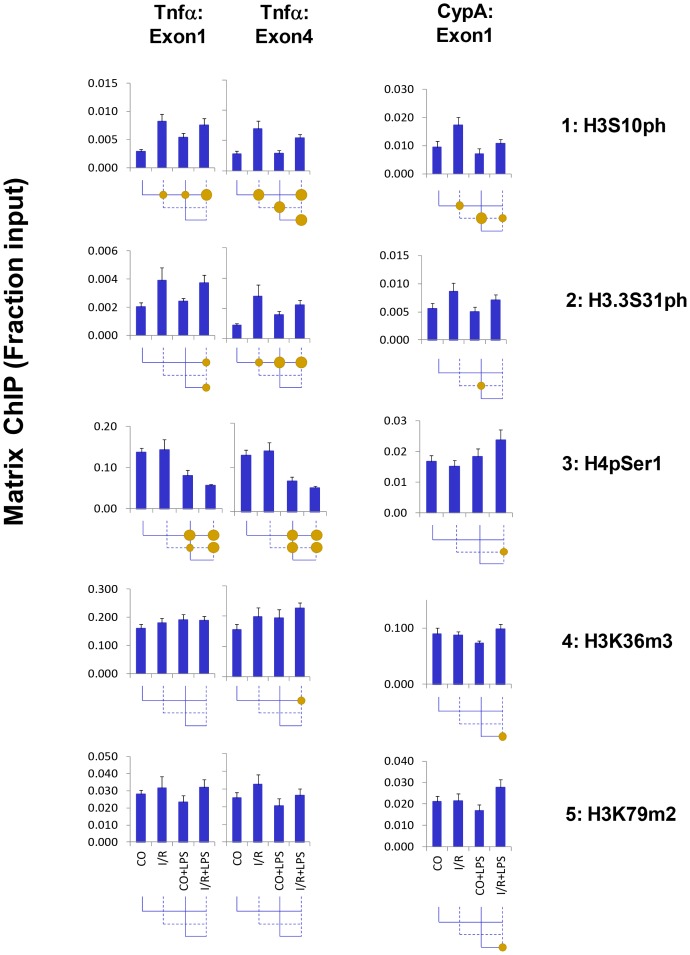
Matrix ChIP analysis of histone marks changes associated with transcription elongation at the *Tnf-α* genes following unilateral kidney I/R and LPS injection. Sheared cross-linked renal cortex chromatin from mice were assayed using antibodies to serine phosphorylated and lysine methylated histone H3. ChIP DNA were analyzed at the Tnf-α first and last exon and CypA first exon in real-time PCR. Data represent mean ± SEM (6 animals from each group), expressed as fraction of input.

Phosphorylation of H3 serine 10 (H3pSer10) is thought to act synergistically with histone acetylation in bringing p-TEFb elongation complex to transcribed genes [37], [38]. I/R increased H3pSer10 levels at the Tnf-α gene but in contrast to histone acetylation, LPS had no effect on this mark (Fig. 4, *row 1*). I/R, but not LPS, also increased H3pSer10 at the CypA gene. The histone variant H3.3 is enriched at actively transcribed genes [39]. H3.3 serine 31 phosphorylation (H3.3pSer31) is also associated with transcription elongation. The pattern of H3.3pSer31 changes in response to injury and LPS was similar to H3pSer10, which is consistent with the fact that the same kinases target both of these sites [23]. Therefore, activation of these histone H3 phosphorylation signaling pathways at the Tnf-α locus is specific to I/R. Histone H4 serine-1 phosphorylation (H4pSer1) is enriched in compacted chromatin and is inversely related to permissive histone H4K8 acetylation [40], [41], [42]. Remarkably, LPS but not I/R dephosphorylated H4pSer1 at the Tnf-α gene (Fig. 4, *row 3*). There was little or no change in H4pSer1 at the CypA site.

#### Histone H3 lysine 36 trimethylation (H3K36m3) and lysine 79 dimethylation (H3K79m2)

Higher levels of H3K36m3 have been correlated with enhanced transcription elongation rates [43], [44]. There was a trend for this mark to be higher in the I/R kidney following LPS injection, mirroring changes in total H3 levels (Fig. 4). No significant changes were found in H3K79m2 levels. The lack of measurable effects suggests that H3K36m3 and H3K79m2 associated with transcription elongation are not playing a major role in priming Tnf-α for endotoxin hyperresponsive state.

#### Histone PTMs associated with transcriptional repression

Analysis of H3K9m2, H3K9m3, H3K27m3 and H4K20m3 repressive marks [45] revealed only small increases in H3K9m2/3 in response to LPS but not I/R at the Tnf-α gene at the time points examined (Fig. S2).

The above results (Figs. 3–4 and Fig. S2) together with previous studies about chromatin changes at Tnf-α gene [15], [46] provide evidence that I/R and LPS increase levels of some but not all permissive histone marks with little or no changes in the canonical repressive marks.

### I/R, but not LPS, Increases Recruitment of Kinases to Chromatin Encompassing Tnf-α Gene

Given that Pol II and histones are phosphorylated in our model, protein kinase cascades may play a prominent role in AKI- and LPS-induced transcription changes. Different classes of kinases are being discovered bound to genes [29], [30], [47]. Signaling cascades including Erk1/2, JNK, and p38 kinases have been implicated in mediating AKI [48]. These enzymes can bind to genes [29], [30], [49], [50]. Any one of these cascades or their combinations could be contributing to the endotoxin hyperresponsiveness [47]. We used Matrix ChIP to identify if components of cascades previously implicated in signaling Tnf-α gene as well as those that phosphorylate Pol II CTD and histones are recruited to Tnf-α locus.

**Figure 5:**
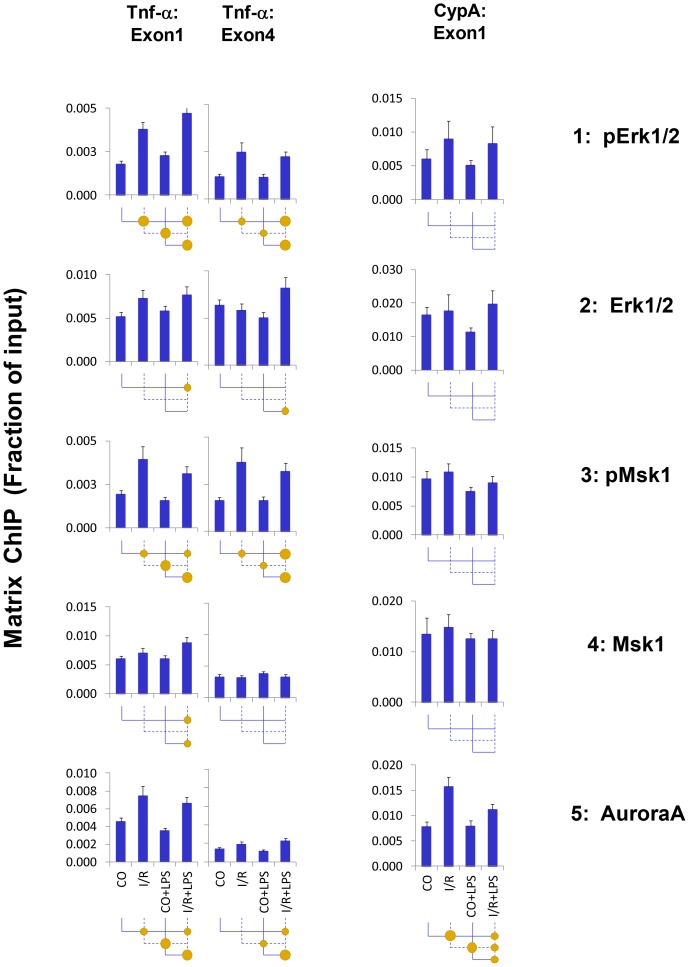
Matrix ChIP analysis of kinases at the *Tnf-α* gene following unilateral kidney I/R and LPS injection. Sheared cross-linked renal cortex chromatin from mice were assayed using antibodies to active (phosphorylated) and total kinases. ChIP DNA were analyzed at the Tnf-α first and last exon and CypA first exon first in real-time PCR. Data represent mean ± SEM (6 animals from each group), expressed as fraction of input.

Msk1/2, activated by Erk1/2, is one of the enzymes that phosphorylate serine 10 of histone H3 [51]. I\R increased levels of active phosphorylated Erk1/2 and Msk1/2 at the Tnf-α gene, but LPS had little or no effect (Fig. 5). Total levels of these kinases remained mostly unchanged, suggesting that these enzymes are constitutively bound to Tnf-α gene and are activated *in situ* [29], [30]. Thus, the patterns of active Msk1/2 and Erk1/2 kinases are similar to H3pSer10/H3.3pS31 profiles at the Tnf-α gene (Fig. 4 & 5).

Next we tested binding of two other H3pSer10/H3.3pS31 kinases, Aurora and IκB kinase α (IKKα). While the Aurora A pattern was similar to H3pSer10 and H3.3pSer31 (Fig. 5), the IKKα pattern resembled Pol II (discussed below).

### I/R and LPS-induced Recruitment of Epigenetic Modifiers to Tnf-α Gene

**Figure 6:**
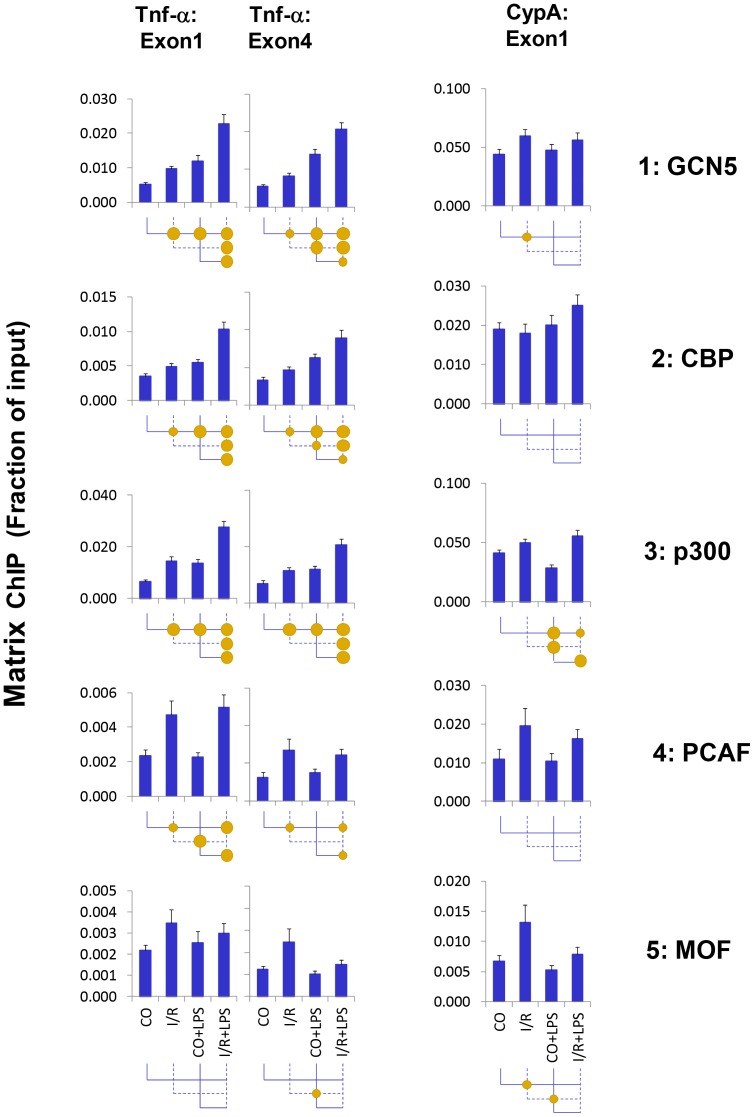
Matrix ChIP analysis of histone acetyltransferases at the *Tnf-α* gene following unilateral kidney I/R and LPS injection. Sheared cross-linked renal cortex chromatin from mice were assayed using antibodies to histone acetyltransferase. ChIP DNA were analyzed at the Tnf-α first and last exon and CypA first in real-time PCR. Data represent mean ± SEM (6 animals from each group), expressed as fraction of input.

I/R and LPS increased histone acetylation of all four histones at the Tnf-α gene (Fig. 3). These PTMs are catalyzed by histone acetyltransferses (HAT). Five classes of HATs are known: p300/CBP, GCN5/PCAF, MYST/MOF, general transcription factors (e.g. TAF1) and nuclear hormone receptor-related [52]. Using Matrix ChIP we identified five HATs – GCN5, CBP, p300, PCAF, and MOF at the Tnf-α gene (Fig. 6). Similar to the Pol II pattern, both I/R and LPS increased GCN5, CBP and p300 levels at the Tnf-α locus, and there was endotoxin hyperresponse. I/R, but not LPS, increased PCAF levels at the Tnf-α gene. Although the difference were not statistically significant similar pattern was seen for MOF. Based on this analysis, it is plausible that hyperacetylation of histone H3K9/14Ac in I/R-LPS kidneys is mediated by either GCN5, p300, CBP or all three acetyltransferases which target one or more of these and other histone H3 lysine residues [53].

**Figure 7:**
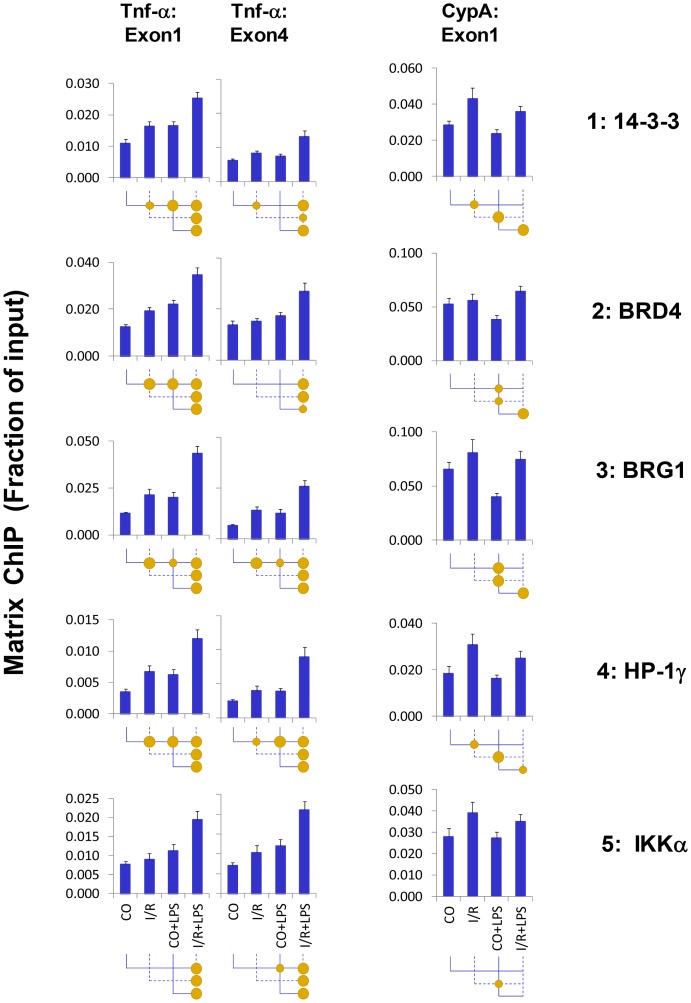
Matrix ChIP analysis of chromatin modifiers at the *Tnf-α* gene following unilateral kidney I/R and LPS injection. Sheared cross-linked renal cortex chromatin from mice were assayed using antibodies to chromatin modifiers. ChIP DNA were analyzed at the Tnf-α first and last exon and CypA first exon in real-time PCR. Data represent mean ± SEM (6 animals from each group), expressed as fraction of input.

Histone H3 acetylation and phosphorylation were both induced at Tnf-α by renal injury (Figs. 3&4), consistent with previous mechanistic studies demonstrating a link between these two histone PTMs [24], [25], [37], [54]. From flies to mammals, the adaptor protein 14-3-3 is tethered to adjacent phosphorylated and acetylated histone H3 residues to facilitate transcription [24], [25], [26], [37]. Its action provides means to synergize histone phosphorylation and acetylation epigenetic pathways [25]. We next tested if 14-3-3 is bound to Tnf-α locus (Fig. 7). Both I/R and LPS increased 14-3-3 levels at the Tnf-α locus, and there was a hyperresponse in LPS-I/R samples. The 14-3-3 pattern at the Tnf-α locus resembled Pol II and H3K9/14, a binding profile consistent with 14-3-3′s role in transcription [24], [37].

The 14-3-3 family of proteins brings several chromatin factors to transcribed loci, including bromodomain containing factors involved in transcriptional elongation [37], [55]. 14-3-3 binding of the bromodomain protein BRD4 is particularly interesting because it recruits the elongation factor P-TEFb to chromatin and as a kinase, BRD4 phosphorylates CTD [37], [55], [56], [57]. The BRD4 binding pattern to Tnf-α gene resembled Pol II and 14-3-3 recruitment, consistent with its role in tethering BRD4 to chromatin [37].

We have previously shown that BRG1 binds to the Tnf-α gene and activates its expression in renal ischemia [46]. Because BRG1 exists in a complex with 14-3-3, we examined BRG1 binding to the gene in response to LPS. The degree of LPS-induced BRG1 recruitment to the Tnf-α gene was similar to that induced by I/R, and there was hyperresponsiveness to LPS in the setting of I/R. Overall the BRG1 binding to Tnf-α gene resembled 14-3-3 and Pol II.

BRG1 interacts with heterochromatin proteins (HP) that regulate chromatin structure [58]. At least one of them, HP-1γ regulates transcription elongation. HP-1γ serves, among other functions, to recruit IKKα to Pol II CTD [23]. I/R and LPS increased levels of HP-1γ at the Tnf-α gene, which were further increased in I/R-LPS kidneys. IKKα is recruited to genes and, among other targets, phosphorylates H3Ser10 [59], [60]. I/R and/or LPS induced IKKα recruitment to Tnf-α gene in a pattern resembling Pol II, 14-3-3, BRG1, and HP-1γ.

## Discussion

We set out to define transcription and chromatin bases and factors to explain augmented expression of endotoxin-inducible Tnf-α gene in AKI. Herein, we uncovered previously unknown genomic events associated with renal Tnf-α gene transcription induced by endotoxin and I/R. PTMs, Pol II and chromatin modifiers that bind to Tnf-α gene can be approximately grouped into five synchronous recruitment patterns (Fig. S3, Type I-V). Type I response exhibited endotoxin hyperresponsiveness, Type II was generated by I/R but not LPS, Type III was a non-additive response to LPS and I/R, and Type IV and V were LPS-predominant responses. Our results suggest that I/R and LPS pathways that induce Tnf-α gene in AKI overlap but are not the same and cooperate to generate a hyperresponse.

### Common Epigenetic Events at the Tnf-α Locus Induced by Either I/R or LPS

We identified a group of chromatin modifiers at the Tnf-α locus whose levels were increased equally high by either LPS or I/R. This set included GCN5, CBP, p300, BRD4, BRG1, HP-1γ and IKKα and their binding patterns resembled Pol II and 14-3-3. CTD serine 7 phosphorylation and H3K9/14 acetylation were also seen with either I/R or LPS. These observations suggest that the same signaling pathways were engaged by both treatments to induce these synchronous events. Bacterial lipopolysaccharides, such as LPS, signal through binding to Toll-like receptor 4 (TLR4), and bacterial lipopeptides signal through TLR2 [61]. TLR4 signaling involves MyD88-dependent (IRAKs/TRAF6) and MyD88-independent (TRIF/TRAF3/RIP1) pathways [62]. TLR2 activation is also transmitted by MyD88 but does not use the TRIF/TRAF3/RIP1 pathway. Leemans et al [63] and Wu et al [64] demonstrated that knock-out of either TLR2, TLR4 or MyD88 prevented renal I/R injury. In I/R-induced renal injury, TLR2 and TLR4 are activated by cell-derived agonists termed damage-associated molecular pattern molecules (DAMPs) [65]. Thus, the same epigenetic events at the Tnf-α gene triggered by either I/R or LPS could be transmitted by MyD88, which receives signals from either activated TLR4 or TLR2.

### Epigenetic Changes at the Tnf-α Locus Specific to Either I/R or LPS

Ramesh et.al. has previously shown that treatment with nephrotoxic cisplatin but not LPS activated MAPK pathways in cultured mouse proximal tubule cells [11]. Similarly, we also found evidence that I/R and LPS activate signaling pathways that are specific to either one of the treatments.

I/R but not LPS increased H3pSer10 and H3.3pSer31 (Fig. 4). In agreement, activities of candidate kinases that target these residues were also increased by I/R only, including Erk1/2, Msk1/2 (which is activated by Erk1/2 [66]) and Aurora-A kinases [67] (Fig. 5). I/R-mediated activation of Erk1/2 depends on TRL2 but not TRL4 [68]. Thus, the I/R-specific epigenetic changes at the Tnf-α gene could be activated by TLR2 in response to released DAMPs.

We also found changes that were induced more strongly by LPS than by I/R including Pol II phosphorylation of CTD serine 2 and 5 (Fig. 2). Histone H4 phosphorylation at Ser 1, H4pSer1, is associated with chromatin compaction and has been shown to oppose histone acetylation, H4K8Ac [41]. In agreement, we found that LPS but not I/R decreased H4pSer1 levels, showing pattern inverse to that of H4K5/8/12/16Ac (Fig. 3, *row 2* and Fig. 4, *row2)*. Different TLR4 agonists can differentially activate Tnf-α expression in the same cell [69] through either MyD88-dependent or MyD88-independent pathways [69]. It is conceivable that some of these pathways are activated more potently by LPS than by I/R triggered TLR4 agonists (e.g. DAMPs), accounting for the differential responses to LPS vs. I/R.

### Synergistic Activation of Tnf-α Gene Expression by I/R and LPS

In AKI, proximal tubules are a major source of cytokine production contributing to renal injury [11], [14]. Mechanistic studies done in diverse systems [23], [24], [25], [37], [54] in conjunction with the current observations showing differential phosphorylation/acetylation responses to I/R and LPS provide the means to piece together a partial view of pathways that cause hyper-activation of Tnf-α transcription by endotoxin in the setting of I/R. We suggest one possible scenario.

We found signaling pathways differentially activated by I/R and LPS at the Tnf-α gene. I/R could initiate cascades that render chromatin encompassing the Tnf-α locus more transcription-permissive (e.g. increased histone phosphorylation/acetylation) and cause recruitment of Pol II to the Tnf-α promoter [46]. The acetyltransferase GCN5 synergistically couples phosphorylation and acetylation epigenetic pathways because its efficiency of acetylation is enhanced by prior histone H3 phosphorylation [54]. Binding of the adaptor protein 14-3-3 to chromatin is essential for Pol II elongation [25], [37]. In the setting of I/R, endotoxin-induced 14-3-3 interaction with chromatin is rendered more stable because of the I/R-induced histone phosphorylation and the additional histone acetylation induced by LPS [24], [37]. As a result, I/R exaggerates LPS-induced recruitment of BRG1 and BRD4 via 14-3-3. While BRG1 loosens nucleosomes and opens access to chromatin [70], BRD4 recruits elongation factors, enhancing Pol II processivity [37]. HP-1γ, which binds to both elongating Pol II as well as histones [23], guides the recruitment of the histone chaperone complex FACT, which facilitates chromatin transcription [22]. In addition, HP-1γ recruits IKKα [23] and other chromatin-associated proteins that facilitate transcription elongation [71]. Dephosphorylation of histone H4 serine-1 facilitates acetylation of H4K8 [41]. Thus, LPS-mediated decrease in H4pSer1 levels (Fig. 4, *row 3*) could also be also contributing to the endotoxin hyperresponse, We suggest that combination of histone phosphorylation and acetylation changes induced by I/R (histone/CTD phosphorylation and acetylation) and LPS (CTD phosphorylation histone acetylation and H4 serine-1 dephosphorylation) (Figs. 3, 5–7) could represent chromatin modifications that interact and contribute to endotoxin Pol II hyperresponsiveness [25], [54]. Thus, it is conceivable that signaling through TLR2 and TLR4 could account for the differential activation of I/R- and LPS-mediated events at the Tnf-αlocus. Studies in TLR2, TLR4 and MyD88 deficient mice will be needed to define the contribution of these receptors and MyD88 in AKI epigenetically mediated endotoxin hyper-responsiveness.

Tnf-α mRNA stability is tightly regulated [15], [72], [73], [74]. For example, we have previously shown that LPS stabilizes Tnf-α mRNA in proximal tubules harvested from LPS-treated mice [15]. Like I/R, cisplatin induces AKI, in part, by increasing proximal tubule Tnf-α production [11], [72]. Ramesh et.al. provided evidence that increased transcription, mRNA stability and translation all contribute to cisplatin induced Tnf-α production [11], [19], [72]. We have found that in the setting of I/R LPS induced >4-fold increase in Tnf-α mRNA levels (Fig. 1) which was greater than the 1.5-2-fold increase in Pol II densities at this locus (Fig. 2). This comparison suggests that changes in mRNA stability may also contribute to the synergistic activation of Tnf-α mRNA expression in our model.

Endotoxin hyperresponsiveness of cytokine production in AKI has been known for nearly a decade [14] but the epigenetic basis for this phenomenon has remained unknown. We identified AKI-induced changes in Pol II and chromatin that exhibit distinct synchronous patterns at the synergistically activated Tnf-α gene. These patterns represent separate signaling cascades that drive expression of Tnf-α and thus provide an epigenetic framework to account for hyperresponsiveness. There are questions that remain unanswered. What is the role of TLR2 and TLR4 in mediating the different I/R and LPS signaling pathways that converge at the Tnf-a locus to induce hyperresponse? What are the DNA regulatory elements and transcription factors that render Tnf-α but not CypA, locus endotoxin hyperresponsive? What other factors are involved that represent other classes of enzymes such as phosphatases, deacetylases and others? What are the hierarchal cause-effect relationships of the events described here? Are the responses same or different for other inflammatory mediators? The general approach used in the current study is well suited to answer these and other relevant questions. The translational application of epigenetic studies of this type is that knowledge of interactions between enzymes bound to relevant genes identifies targets for potential drug intervention to ameliorate renal injury.

## Methods

### Reagents

Bovine serum albumin (BSA), phosphate buffered saline (PBS), salmon sperm DNA, and protein A were from Sigma, and proteinase K was from Invitrogen. Matrix ChIP-MeDIP 96-well polypropylene plates were from Bioexpress. Formaldehyde, ethanol, NaCl, EDTA, Triton X-100, NP-40, Tris, leupeptin, PMSF, p-nitrophenyl phosphate, NaF, Na_3_VO_4_, Na_2_MoO_4_ and β-glycerophosphate were from Sigma. The antibodies were commercially available and are listed in Table S2.

### In vivo Ischemia-reperfusion (I/R) Protocol

The experimental protocol is shown in Fig. S1. Male CD 1 mice (Charles River Laboratories, Wilmington, MA; 6–10 weeks of age; 30–35 gms), maintained under routine vivarium conditions. In vivo experiments involved a mouse model of kidney injury. The specific protocol used in this study was approved by The Institutional Animal Care and Use Committee (IACUC) at the University of Washington. In brief, mice were anesthetized and subjected to a midline abdominal incision under sterile conditions. Left renal ischemia was induced with an atraumatic microvascular clamp applied to the renal pedicle. After 30 min of unilateral renal artery occlusion, the clamp was released and reperfusion of the entire kidney was assessed visually (by loss of global cyanosis). A 30 min ischemic insult was selected for study because it induces moderately severe ischemic kidney damage. Twenty-four hrs after I/R injury, mice received a tail vein injection of either LPS or saline. Two hours after injection, mice were deeply anesthetized, the abdominal cavity was opened, the kidneys were extracted, and renal cortical samples were cut from the kidneys with a razor blade and rapidly frozen at −70°C. As previously documented [14] the right non-ischemic, contralateral (control) kidney recapitulates what is seen in sham operated kidneys and hence served as an internal control.

### RNA Extraction and cDNA Synthesis

RNA was extracted from tissue fragments using Trizol reagent as per the manufacturer’s protocol. To synthesize cDNA, 400 ng of Trizol-extracted total RNA was reverse transcribed with 200 units MMLV reverse transcriptase (Invitrogen) and random hexamer primers in 20 µl reactions. RT reactions were diluted 100-fold prior to running qPCR [75].

### Chromatin Preparation and Multiplex Matrix ChIP Platform

The multiplex microplate Matrix ChIP method was previously described [28]. Briefly, for ChIP assays, tissue fragments (10–20 mg) were cross-linked with formaldehyde, and chromatin was sheared using Diagenode Bioruptor [28]. ChIP assays were done using protein A-coated 96-well polypropylene microplates as described before. 1–2 µl of eluted DNA was used in 2–4 µl real-time PCR reactions (ABI7900HT). All PCR reactions were run in quadruplicates. PCR calibration curves were generated for each primer pair from a dilution series of total mouse or human genomic DNA. The PCR primer efficiency curve was fit to cycle threshold (Ct) versus log(genomic DNA concentration) using an r-squared best fit. DNA concentration values for each ChIP and input DNA sample were calculated from their respective average Ct values. Final results are expressed as fraction of input DNA [31]. Matrix ChIP PCR primers are shown in Table S1 and list of antibodies in Table S2.

### Statistics and Visualization

To acquire, store and analyze large data sets generated by the high throughput Matrix ChIP platform, we developed a novel graphical method (GraphGrid). Pair-wise statistically significant differences are represented by the size of a circle for each comparison made; p<0.05 by small circle, p<0.01 by large circle and no circle indicating non-significance. The tool automatically adjusts the computed pairwise p-value (significance t-test) using a Bonferroni correction factor (Fig. S1B–C).

Additional information about the methods and data are available upon request.

## Supporting Information

Figure S1 **Ischemia-reperfusion and endotoxin acute renal injury model and data analysis. A.** ***Model.*** Mice were anesthetized and subjected to a midline abdominal incision under sterile conditions and, after 30 min of unilateral renal artery occlusion, the clamp was released (ischemia/reperfusion, or I/R). Twenty-four hours later, I/R injury mice received a tail vein injection of either lipopolysaccharide (LPS) or saline [14]. Two hours after injection, mice were anesthetized, and kidneys were harvested and rapidly frozen for RT-PCR and Matrix ChIP analysis [28], [31]. Two hours post was chosen because the peak response to LPS is seen at this time. **B.** ***GraphGrid analysis.*** Results of statistical analysis of endotoxin and I/R responses are shown. Graph bars represent mean values±SEM. Solid yellow circles positioned at line intersections below the graph designate significant differences between given pairs of means. Bar above the circle represents one of the paired means. The second of the paired means is located above the very left end of horizontal line crossing the yellow circle. Five different paired statistical comparisons are done as shown with the numbered circles (right panel). **C**. Statistical analysis is done using Bonferroni correction. Statistical differences between two means (p value) are shown by the size of the solid yellow circle. : p<0.05 by small circle, p<0.01 by large circle, and no circle indicating the differences are not statistically significant.
(TIF)

Figure S2 **Matrix ChIP analysis of repressive histone lysine methylated marks at the** ***TNF-α*** **genes following unilateral kidney I/R and LPS injection.** Sheared cross-linked renal cortex chromatin from mice were assayed using antibodies to histone H3 and H4 lysine methylated residues. ChIP DNA were analyzed at the Tnf-α first and last exon and CypA first exon in real-time PCR. Data represent mean ± SEM (6 animals from each group), expressed as fraction of input.
(TIF)

Figure S3 **Synchronized epigenetic changes grouped into different types of responses to I/R and LPS treatment in AKI.** *Type I*, I/R and LPS co-responsive/hyperresponsive; *Type II,* I/R only responsive; *Type III*, I/R and LPS co-responsive at saturation; and *Type IV and V*, LPS only responsive.
(TIF)

Table S1 **Sequences of primers used in ChIP-qPCR primers.**
(DOCX)

Table S2 **Antibodies used in Matrix ChIP assays.**
(DOCX)
