# Supplementary figures and images for: Synchronous Recruitment of Epigenetic Modifiers to Endotoxin Synergistically Activated Tnf-α Gene in Acute Kidney Injury
Source: PLoS One. 2013 Jul 30;8(7):e70322. doi: 10.1371/journal.pone.0070322 (PMC3728219; doi:10.1371/journal.pone.0070322)

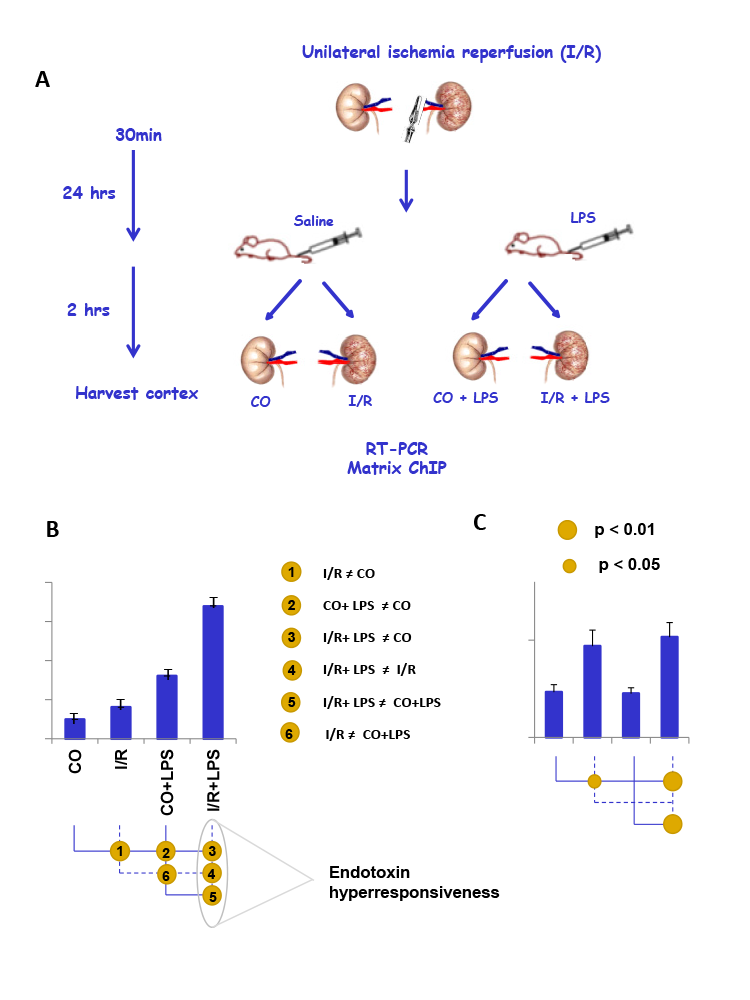

Supplement: Figure S1 — Ischemia-reperfusion and endotoxin acute renal injury model and data analysis. A. Model. Mice were anesthetized and subjected to a midline abdominal incision under sterile conditions and, after 30 min of unilateral renal artery occlusion, the clamp was released (ischemia/reperfusion, or I/R). Twenty-four hours later, I/R injury mice received a tail vein injection of either lipopolysaccharide (LPS) or saline [14]. Two hours after injection, mice were anesthetized, and kidneys were harvested and rapidly frozen for RT-PCR and Matrix ChIP analysis [28], [31]. Two hours post was chosen because the peak response to LPS is seen at this time. B. GraphGrid analysis. Results of statistical analysis of endotoxin and I/R responses are shown. Graph bars represent mean values±SEM. Solid yellow circles positioned at line intersections below the graph designate significant differences between given pairs of means. Bar above the circle represents one of the paired means. The second of the paired means is located above the very left end of horizontal line crossing the yellow circle. Five different paired statistical comparisons are done as shown with the numbered circles (right panel). C. Statistical analysis is done using Bonferroni correction. Statistical differences between two means (p value) are shown by the size of the solid yellow circle. : p<0.05 by small circle, p<0.01 by large circle, and no circle indicating the differences are not statistically significant. (TIF) [file pone.0070322.s001.tif]

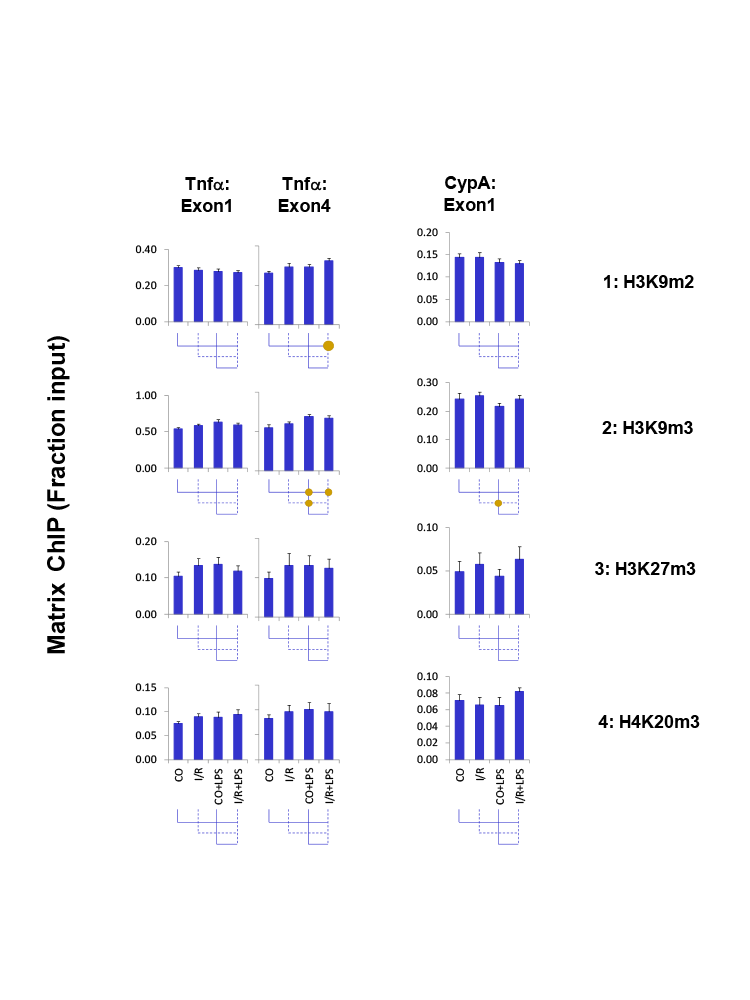

Supplement: Figure S2 — Matrix ChIP analysis of repressive histone lysine methylated marks at the TNF-α genes following unilateral kidney I/R and LPS injection. Sheared cross-linked renal cortex chromatin from mice were assayed using antibodies to histone H3 and H4 lysine methylated residues. ChIP DNA were analyzed at the Tnf-α first and last exon and CypA first exon in real-time PCR. Data represent mean ± SEM (6 animals from each group), expressed as fraction of input. (TIF) [file pone.0070322.s002.tif]

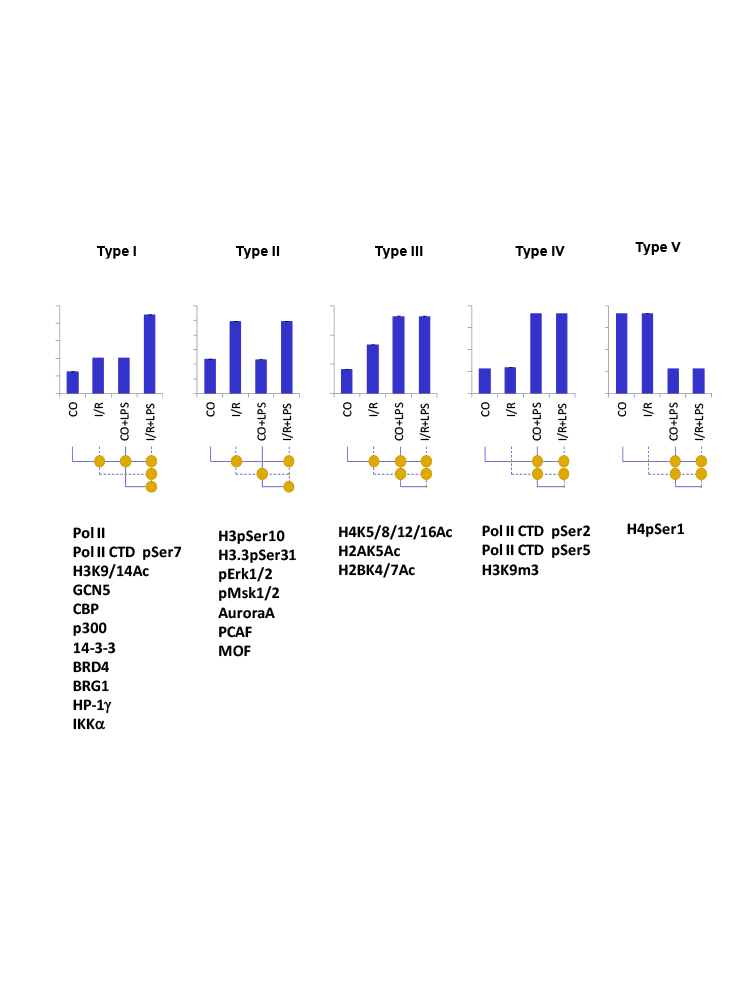

Supplement: Figure S3 — Synchronized epigenetic changes grouped into different types of responses to I/R and LPS treatment in AKI. Type I, I/R and LPS co-responsive/hyperresponsive; Type II, I/R only responsive; Type III, I/R and LPS co-responsive at saturation; and Type IV and V, LPS only responsive. (TIF) [file pone.0070322.s003.tif]
