# Supplementary material for: Synchronous Recruitment of Epigenetic Modifiers to Endotoxin Synergistically Activated Tnf-α Gene in Acute Kidney Injury
Source: PLoS One. 2013 Jul 30;8(7):e70322. doi: 10.1371/journal.pone.0070322 (PMC3728219; doi:10.1371/journal.pone.0070322)
Supplement: Table S1 — Sequences of primers used in ChIP-qPCR primers. (DOCX) [file pone.0070322.s004.docx]

**Table S1. Sequences of primers used in ChIP-qPCR**

| Primer | Sequence (all mouse) |
| --- | --- |
| Tnf-α Exon1 FWD | AGTGCCTCTTCTGCCAGTTC |
| Tnf-α Exon1 REV | GCAGGTTCTGTCCCTTTCAC |
| Tnf-α Exon4 FWD | GCTCCAGTGAATTCGGAAAG |
| Tnf-α Exon4 REV | TATGGCTCAGGGTCCAACTC |
| CypA Exon1 FWD | CGTTTGGAAAGCAGTTGTGA |
| CypA Exon1 REV | AGAACACGGTGGGGTTGAC |
| β-Actin FWD | TCTCAGCTGTGGTGGTGAAG |
| β-Actin REV | GTCCCTGTATGCCTCTGGTC |
