# Supplementary material for: Synchronous Recruitment of Epigenetic Modifiers to Endotoxin Synergistically Activated Tnf-α Gene in Acute Kidney Injury
Source: PLoS One. 2013 Jul 30;8(7):e70322. doi: 10.1371/journal.pone.0070322 (PMC3728219; doi:10.1371/journal.pone.0070322)
Supplement: Table S2 — Antibodies used in Matrix ChIP assays. (DOCX) [file pone.0070322.s005.docx]

**Table S2. Antibodies used in Matix ChIP**

| Figure | Row | Antibody | Catalog No | Source | Manufacturer |
| --- | --- | --- | --- | --- | --- |
| Primary | | | | | |
| 2 | 1 | Pol II CTD (4H8) | sc-47701 | Mouse monoclonal | Santa Cruz |
| 2 | 2 | Pol II (N-20) | sc-899 | Rabbit polyclonal | Santa Cruz |
| 2 | 3 | Pol II CTD pSer7 | 61088 | Rat monoclonal | Active Motif |
| 2 | 4 | Pol II CTD (H14) pSer5 | MMS-134R | Mouse monoclonal IgM | Covance |
| 2 | 6 | Pol II CTD (H5) pSer2 | MMS-129R | Mouse monoclonal IgM | Covance |
| 2 | 7 | Pol II CTD (8WG18) | MMS-126R | Mouse monoclonal | Covance |
| 3 | 1 | H3K9/14Ac | 06-599 | Rabbit polyclonal | Millipore |
| 3 | 2 | H4K5/8/12/16Ac | 06-866 | Rabbit serum | UPSTATE |
| 3 | 3 | H2AK5Ac | ab1764 | Rabbit serum | ABCAM |
| 3 | 4 | H2B4/7Ac | ab1759 | Rabbit serum | ABCAM |
| 3 | 5 | H3 | ab1791 | Rabbit polyclonal | ABCAM |
| 4 | 1 | H3pSer10 | ab14955 | Mouse monoclonal | ABCAM |
| 4 | 2 | H3.3pSer31 | ab2889 | Rabbit polyclonal | ABCAM |
| 4 | 3 | H4pSer1 | PA5-27064 | Rabbit polyclonal | ThermoPierce |
| 4 | 4 | H3K36m3 | ab9050 | Rabbit polyclonal | ABCAM |
| 4 | 5 | H3K79m2 | ab3594 | Rabbit polyclonal | ABCAM |
| 5 | 1 | pErk1/2 | #4370 | Rabbit monoclonal | Cell Signaling |
| 5 | 2 | Erk1 (K-23) | sc-94 | Rabbit polyclonal | Santa Cruz |
| 5 | 2 | Erk1 (K-23) | sc-153 | Rabbit polyclonal | Santa Cruz |
| 5 | 3 | pMsk1 (Thr581) | #9595 | Rabbit polyclonal | Cell Signaling |
| 5 | 4 | Msk1 (H-65) | sc-25417 | Rabbit polyclonal | Santa Cruz |
| 5 | 5 | AuroraA (35C1) | MA1-80399 | Mouse monoclonal | ThermoPierce |
| 6 | 1 | GCN5 | PA5-30041 | Rabbit polyclonal | ThermoPierce |
| 6 | 2 | CBP (A-22) | sc-369 | Rabbit polyclonal | Santa Cruz |
| 6 | 3 | p300 (N-15) | sc-584 | Rabbit polyclonal | Santa Cruz |
| 6 | 4 | PCAF (E.107.0) | MA-11186 | Mouse monoclonal | ThermoPierce |
| 6 | 5 | MOF/MYST1 | A300-994A-1 | Rabbit polyclonal | Bethyl |
| 7 | 1 | 14-3-3 (K-19) | sc-629 | Rabbit polyclonal | Santa Cruz |
| 7 | 2 | BRD4 | ARP39076 | Rabbit polyclonal | Aviva |
| 7 | 3 | BRG1/SNF2β | 07-478 | Rabbit serum | UPSTATE |
| 7 | 4 | HP-1γ | 05-690 | Mouse monoclonal | UPSTATE |
| 7 | 5 | IKKα (B−8) | sc -7606 | Mouse monoclonal | Santa Cruz |
| S2 | 1 | H4K20m3 | 07-463 | Rabbit polyclonal | Millipore |
| S2 | 2 | H3K27m3 | ab6002 | Mouse monoclonal | ABCAM |
| S2 | 3 | H3K9m2 | Ab1220 | Mouse monoclonal | ABCAM |
| S2 | 4 | H3K9m3 | Ab8898 | Mouse monoclonal | ABCAM |
| Secondary | | | | | |
| 2 | 3&4 | Anti-Mouse IgM μ chain | 315-005-020 | Rabbit polyclonal | JacksonImmunoresearch |
| 2 | 2 | Anti-Rat IgG | 31218 | Rabbit polyclonal | ThermoPierce |
